# Chronodisruption and Ambulatory Circadian Monitoring in Cancer Patients: Beyond the Body Clock

**DOI:** 10.1007/s11912-021-01158-z

**Published:** 2022-01-21

**Authors:** Pedro F. Almaida-Pagan, María Torrente, Manuel Campos, Mariano Provencio, Juan Antonio Madrid, Fabio Franco, Beatriz Rodríguez Morilla, Blanca Cantos, Pedro A. Sousa, María José Martínez Madrid, Joao Pimentao, María Ángeles Rol

**Affiliations:** 1 Kronohealth SL, Murcia, Spain; 2 Chronobiology Lab, Department of Physiology, College of Biology, University of Murcia, Mare Nostrum Campus, IUIE, IMIB-Arrixaca, Murcia, Spain; 3 Ciber Fragilidad Y Envejecimiento Saludable (CIBERFES), Instituto de Salud Carlos III, Madrid, Spain; 4 Servicio de Oncología Médica, Hospital Universitario Puerta de Hierro-Majadahonda, Madrid, Spain; 5 Medical Oncology Department, Puerta de Hierro-Majadahonda University Hospital, Calle Manuel de Falla, 1, 28222 Madrid, Spain; 6 Faculty of Health Sciences, Francisco de Vitoria University, Madrid, Spain; 7 Department of Electrical Engineering, Faculty of Science and Technology, Universidade Nova de Lisboa, Lisbon, Portugal

**Keywords:** Circadian rhythms, Chronodisruption, Ambulatory circadian monitoring, Cancer patients, Tumorigenesis, Multi-modal sensors

## Abstract

**Purpose of Review:**

Circadian rhythms impose daily rhythms a remarkable variety of metabolic and physiological functions, such as cell proliferation, inflammation, and DNA damage response. Accumulating epidemiological and genetic evidence indicates that circadian rhythms’ disruption may be linked to cancer. The integration of circadian biology into cancer research may offer new options for increasing cancer treatment effectiveness and would encompass the prevention, diagnosis, and treatment of this disease.

**Recent Findings:**

In recent years, there has been a significant development and use of multi-modal sensors to monitor physical activity, sleep, and circadian rhythms, allowing, for the very first time, scaling accurate sleep monitoring to epidemiological research linking sleep patterns to disease, and wellness applications providing new potential applications.

**Summary:**

This review highlights the role of circadian clock in tumorigenesis, cancer hallmarks and introduces the state-of-the-art in sleep-monitoring technologies, discussing the eventual application of insights in clinical settings and cancer research.

## Introduction: on the Clock

Recent studies in circadian biology bear Benjamin Franklin, the sage of colonial America, out, on his advice “Early to bed and early to rise, makes a man healthy, wealthy, and wise.” Staying in synchrony with the 24-h light–dark cycle of Earth has indeed proven benefits to human health and brain function [1].

Circadian biology studies the biochemical clocks that keep time in our brains and most cells in our bodies. Evidence is accumulating that misalignment of these clocks with the daily light–dark cycle of our environment can have profound impact on physiology, raising the risk of disease. Circadian rhythms control a remarkable variety of metabolic and physiological functions in the human body. Accumulating epidemiological and genetic evidence in the last years indicates that the disruption of circadian rhythms, or chronodisruption (CD), may be directly linked to cancer, due to impairment of cellular functions important for tumor suppression including cell proliferation, senescence, metabolism, and DNA damage response (DDR) [1, 2••, 3]. Perturbations of these processes are hallmarks of cancer and chronic circadian rhythm disruption predisposes to tumor development. Indeed, several molecular gears constituting the circadian clock machinery have been found to establish functional interplays with regulators of the cell cycle, and alterations in clock function could lead to aberrant cellular proliferation [4]. In addition, connections between the circadian clock and cellular metabolism, regulated by chromatin remodeling, have been identified, thus suggesting that abnormal metabolism in cancer could also be a consequence of a disrupted circadian clock [3, 5••].

Circadian rhythms are endogenously generated rhythms that occur with a periodicity of approximately 24 h. In humans, as in all animals, circadian rhythms regulate hundreds of activities, from sleep patterns to body temperature, or digestion. The machinery of the molecular clock in mammals, thoroughly described in literature [1, 3], is based on auto-regulatory transcriptional feedback loops driven by the heterodimer of transcription factors BMAL1/CLOCK which binds DNA recognition sequences called E-boxes and activates the expression of a wide number of genes (clock-controlled genes or CCGs, representing 3–10% of all mRNAs expressed in any tissue).

Among all CCGs, the heterodimer BMAL1/CLOCK activates the expression of their downstream transcriptional repressor targets *Cryptochrome* (*Cry1, 2*) and *Period* (*Per1, 2, 3*) at the beginning of the circadian day. The accumulation of PER and CRY proteins in the cytoplasm by the end of the circadian day leads to the formation of a PER/CRY repressor complex that translocates into the nucleus at the beginning of the circadian night and inhibits the activity of BMAL1/CLOCK heterodimers. In addition, there is a second feedback loop by which the transcription of *Bmal1* is alternatively regulated by two of its own transcription targets, the nuclear receptors REV-ERBα/β (the repressors) and RORα (the activator) [6]. The multiple interlocked auto-regulatory feedback loops result in a robust circadian variation in the expression and activity of *Bmal1* over a 24-h period, providing a driving force for circadian oscillation of a wide number of genes, some of which are key regulators of cell proliferation, metabolism, senescence, and DDR.

Although circadian rhythms are autonomously generated at the cell level due to the existence of the molecular clock, these rhythms also respond to an internal time, which is set by a master clock or central pacemaker. In humans, this role is played by the suprachiasmatic nucleus (SCN), a hypothalamus region that through physical (temperature swings), humoral (melatonin and cortisol), and nervous signals (autonomic nervous system), synchronizes the peripheral clocks. The SCN is inherently sensitive to environmental cues, so-called *zeitgebers* (German for time-givers). The most powerful *zeitgeber* is the 24-h light/dark cycle, which resets circadian functions by acting on neurons within the SCN via a direct connection with the melanopsin-expressing ganglion cells in the retina [6]. Therefore, environmental light “sets the time” of the central clock or circadian pacemaker, which in turn synchronizes the billions of peripheral clocks located in every single nucleated cell and drives the expression of key genes for cancer development.

A shift in the synchronizing environmental cues, for example, as a consequence of a flight crossing different time zones, or working night shifts, induces a change in the central clock phase that directly affects the peripheral clocks that reside in various tissues throughout the body [7–9]. If these changes become chronic, something that is happening nowadays as a consequence of modern lifestyle, a separation between our biological time (internal time set by the SCN), our habits (social time), and the environmental synchronizers (external time) may take place, which can in turn cause a relevant impairment of the circadian organization of physiology, endocrinology, metabolism, and behavior in the human body. This phenomenon, recently denominated with the term chronodisruption (CD) [10], can manifest as a reduction in the amplitude of circadian rhythms, as a complete asynchrony, an advance, or a delay of the peripheral clocks, compared to the SCN phase or even as a phase inversion of the rhythms [11]. It has been proven that CD is closely linked to chronic processes such as aging and cancer [2••, 5••, 12, 13, 14••, 15, 16•]. Of interesting note, in 2007 (and revised in 2019), the International Agency for Research on Cancer (IARC) from the World Health Organization (WHO) classified the “shift-work that involves circadian disruption,” which includes both working at night and working in a job that involves rapidly crossing many time zones, as probably carcinogenic to humans [17].

## Circadian System Status in Cancer Patients

Alterations caused by cancer itself or by anti-cancer therapy at cell and/or tissue levels may disrupt CS functioning [18, 19]. Cancer, or the organism response to stress caused by cancer, leads to an increased release of pro-inflammatory cytokines that affect the hypothalamus–pituitary–adrenal axis and cause CS deregulation [20]. Impairment of CS correlates to several symptoms in cancer patients, such as reduced capability to cope with daily-life activities, insomnia, appetite loss, and fatigue, all of them diminishing quality of life (QoL) [19, 21–25].

Alternatively, several studies in humans have evidenced that CD may not only be a consequence of tumor development, but also may cause cancer and promote its progression. This has been particularly shown in metastatic colorectal cancer [26–29], in advanced breast cancer [24, 30–36], and in lung cancer [21, 37–39]. Besides, circadian rhythm dysfunction is also linked to lower anti-cancer treatment efficacy and, overall, to reduce survival [22, 23, 27, 29, 31].

## Chronodisruption as Cause of Cancer

Erren and Reiter [10] proposed that CD may play a critical role in the expression and development of disease through two related ways: firstly, as a possible cause of chronic disease by representing the adverse split of a physiological nexus of internal and external times, and secondly, by being a critical link in the chain of causation leading to chronic disease, including cancer [40], via a relevant disturbance of the circadian organization of physiology, endocrinology, metabolism, and behavior.

In this sense, we can define chronodisruptors as exogenous and endogenous “exposures” or “effectors” which are chronobiologically active and can thus disrupt the timing and order, i.e., temporal organization of physiologic functions and hierarchies. In principle, whatever allows the establishment of temporal organizational order in organisms should also be capable of disrupting such order or temporal program when present, or applied in excess or deficit and, most importantly, at unusual and inappropriate times, especially when combined with further agonistic or antagonistic chronobiological effectors [41]. An endogenous chronodisruptor may be a mutation in a clock gene or the alteration in cell signaling as a consequence of tumor development. In this case, the tumor is able to impact the molecular clock functioning and cause CD.

Alternatively, one key exogenous or external chronodisruptor is light at night (LAN). Light, when received at unusual times, can powerfully disrupt the circadian rhythmicity of our biology, thus leading to CD and to an increase in the risk of developing certain diseases. In this case, CD would happen independently of the tumor and would be a risk factor for its genesis and progression. Epidemiologic studies have shown that LAN may contribute to higher breast cancer rates in women with normal sight [42–44], while the opposite occurs in visually impaired women who are insensitive to light changes in the environment, and thus depend largely on free-running endogenous circadian clocks to synchronize daily physiology [45, 46]. Several studies suggest that totally blind women are half as likely to have breast cancer as women who are not totally blind [46]. Thus, further research is needed in order to elucidate the impact on CD in these blind patients and how these factors may relate to breast cancer risk. Besides, working night shifts and/or flying frequently across different time zones may also be associated to increased risk of developing many forms of cancer [47], and melatonin suppression and/or disruption of its adequate timing under chronodisruptive conditions may be an important factor for increasing cancer risk [47–49].

Other exogenous effectors, such as our daily eating time schedule [50] or physical activity [51], that contribute to synchronize peripheral clocks (along with the light/dark cycle), can also be disruptors when performed at inadequate times.

## How to Assess CD: Ambulatory Circadian Monitoring

Recently, several prestigious scientific journals have published about health risks produced by inadequate exposure to synchronizers [52–54] and the need to foster the development and validation of “wearable” devices, m-Health applications, and instruments suitable for population-based and big data approaches to develop predictive models and biomarker platforms for assessing circadian health [55, 56]. As a matter of fact, measuring CD is still an unresolved challenge, especially under free-living conditions. Of note, it has been proposed as a bona fide public health issue in light of the very substantial number of individuals involved [57]. The development of wearable multisensorized devices (i.e., electronic health records) and procedures to accurately quantify sleep and CD, both in clinical and nonclinical environments, is key for personalized medicine through unhealthy habit detection and assisting in the diagnose and treatment of diseases.

Furthermore, the workshop on “Developing Biomarker Arrays Predicting Sleep and Circadian-Coupled Risks to Health” jointly sponsored by the National Heart Lung and Blood Institute, National Institute on Aging, and the Sleep Research Society specifically identified the “gaps” to implement potentially high-yield strategies in biomarker research that can be summarized in Table 1 [55].

**Table 1:** ACM for circadian system status in cancer patients: state of the art

*Cancer type*	*Stage*	*Results*	*Study*	*ACM method*	*Rhythms analyzed*
*Breast cancer*	**General**	Circadian rhythm disruption is involved in the experience of fatigue and depression in cancer patients	[58]	Wrist actigraphy	RAR/SWR
		Significant relationships between subjective and objective sleep, but no consistent patterns	[59]	Wrist actigraphy	RAR/SWR
	**Before CT**	Disturbed sleep and fatigue	[24]	Wrist actigraphy	RAR/SWR
		Short sleep time and long naps	[33]	Wrist actigraphy	SWR
		High number and length of night awakenings	[59]	Wrist actigraphy	RAR/SWR
		Low mean daytime activity			
		RAR disrupted	[59, 60]	Wrist actigraphy	RAR/SWR
		Patients with more delayed rhythms experience more daily dysfunction secondary to fatigue	[24]	Wrist actigraphy	RAR/SWR
		Less recorded nap time correlated with physical component scale of the medical outcomes study 36-item short form	[33]	Wrist actigraphy	SWR
	**During CT**	SWR impaired during the first week of both CT cycles 1 and 4	[36]	Wrist actigraphy	SWR
		RAR parameters decreased with the increasing number of CT cycles	[30, 31, 60]	Wrist actigraphy	RAR/SWR
	**After CT**	Altered RAR parameters at 5 years after the primary diagnosis	[32]	Wrist actigraphy	RAR
	**In-patients**	The hospitalization exacerbated the problems associated with RAR	[31]	Wrist actigraphy	RAR
	**Melatonin**	Bedtime melatonin improved sleep quality, sleep fragmentation, and quantity	[61]	Wrist actigraphy	RAR/SWR
*Lung cancer*	**Before CT**	Poor SWR before treatment	[38]	Wrist actigraphy	SWR
		Lower sleep efficiency and higher sleep fragmentation during the night	[62]	Wrist actigraphy	RAR/SWR
		Lower mean activity during the day			
	**During CT**	Disturbed RAR/SWR in cancer patients	[21, 39, 63]	Wrist actigraphy	RAR/SWR
		Circadian disruption selectively affects specific quality of life domains	[21]	Wrist actigraphy	RAR/SWR
		CD correlated with poor QoL and function	[39, 63]	Wrist actigraphy	RAR/SWR
		Cancer symptoms had a stronger association with sleep than mood	[39]	Wrist actigraphy	SWR
		SWR improves at week 48 after start of treatment	[38]	Wrist actigraphy	SWR
*Colorectal cancer*	**General**	An unstable RAR associates to lower QoL and survival	[22, 26]	Wrist actigraphy	RAR
		RAR associated with serum levels of TGF-alpha, IL-6, cortisol, and tumor-related symptoms	[64]	Wrist actigraphy	RAR
	**Before CT**	RAR disruption before treatment. RAR is good predictor of overall survival and progression-free survival	[65]	Wrist actigraphy	RAR
		CD in 52% patients: lower median activity counts, worse fragmented sleep, and an abnormal temperature rhythm	[66]	Chest sensor	Chest skin temp and activity
	**During CT**	RAR correlated with global QoL, physical functioning, social functioning, fatigue, and appetite loss	[23]	Wrist actigraphy	RAR
		RAR independently predicted for overall survival (OA)			
		CD in 51% patients on CT. Associated to shorter OA	[27]	Wrist actigraphy	RAR
		Higher survival in patients with a robust RAR			
		RAR CD associated with severe fatigue and anorexia	[29]	Wrist actigraphy	RAR
		Interference with physical and social functioning			
*Gastro-intestinal cancer*	**During CT**	Three circadian rhythms and the TAP rhythm grew less stable and more fragmented in response to CT	[61]	Inner wrist surface temp	DST
		Large inter- and intra-individual changes were found for T, A, P, and TAP, with phase differences of up to 12 h among patients		Arm activity	Activity
		A moderate perturbation of temporal internal order was observed		Arm position	Position
*Breast and gynecologic cancers*	**After CT**	Systematic bright light exposure in the morning may have beneficial effects on sleep in fatigued cancer survivors	[67]	Wrist actigraphy	SWR
*Non-cancer specific*	**During CT**	Disruptions in skin temperature rhythms	[68]	Skin surface temperature patches	CBT
	**During CT**	Circadian patterns in skin surface temperature and RAR persisted or were amplified during and after CT in 50% patients	[69]	Skin surface temperature patches	CBT
		In contrast, transient or sustained disruption of these biomarkers was found for the remaining 50%		Wrist actigraphy	RAR
		Large inter-patient differences in circadian amplitudes and acrophases of skin surface temperature despite rather similar RAR acrophases			
	**During CT**	RAR disruption	[70]	Wrist actigraphy	RAR
	**During CT**	Hospitalization alters RAR parameters markedly and deteriorates QoL	[71]	Wrist actigraphy	RAR
	**During CT**	Correlation between questionnaires (subjective) and actigraphy (objective) to estimate sleep	[72]	Wrist actigraphy	SWR
		Awakenings during the sleep period (estimated through ACM) best correlated with subjective sleep complaints			

Abbreviations: *ACM*, ambulatory circadian monitoring; *CBT*, core body temperature; *CD*, chronodisruption; *CT*, chemotherapy; *DST*, distal skin temperature; *IL*, interleukin; *PCS*, physical component scale; *QoL*, quality of life; *RAR*, rest-activity rhythm; *SF-36*, medical outcomes study 36-item short form health survey; *SWR*, sleep–wake rhythm; *TAP*, temperature, activity, and position integrated variable; *TGF*, transforming growth factor

**Fig. 1:**
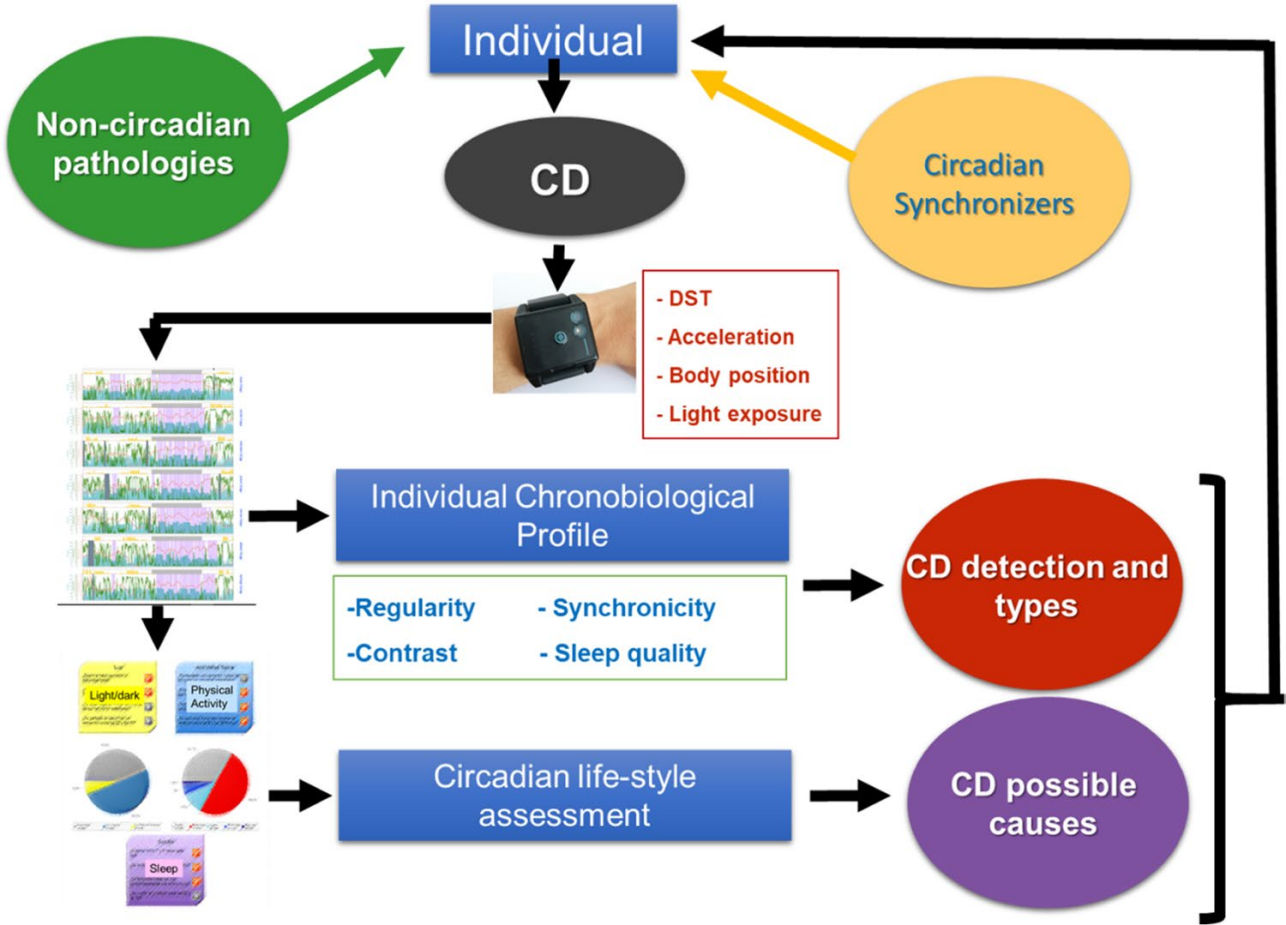
Ambulatory circadian monitoring (ACM) for chronodisruption (CD) detection. ACM, thanks to several sensor implementation in wearable devices (i.e., wristwatch-like), combines measurements of (**a**) endogenous variables, such as distal skin temperature; (**b**) *zeitnehmers*, such as motor activity and body position more dependent on willingness; and (**c**) exogenous synchronizers, such as light exposure and environmental temperature, providing information about lifestyle and the bidirectional crosstalk between internal time and external synchronizers, which is paramount to determine a subject exposure to CD.

Due to its location in the brain, human SCN function or its responses to light can only be indirectly assessed by the so-called circadian marker rhythms [73]. Ambulatory multivariable recordings were for the first time proposed by our group [73, 74] (Fig. 1).

Sensor implementation in wearable devices (i.e., wrist watch-like) allows ACM to combine measurements of (a) endogenous variables, such as skin temperature [75]; (b) *zeitnehmers* (German word meaning timekeeper), such as motor activity and body position more dependent on willingness [76]; and (c) exogenous synchronizers or *zeitgebers*, such as light exposure and environmental temperature, providing information about lifestyle and the bidirectional crosstalk between internal time and external synchronizers [77–79], which is paramount to determine a subject suffering from CD [10]. If the environmental time (given by the light/dark cycle mainly), the internal time (marked endogenously and autonomously by the SCN), and the social time (given by the external interval for work or social activities) are in phase, we should not expect CD. The same will be anticipated when the appropriate phase relationship among overt rhythms (sleep, temperature, activity, etc.) is maintained.

ACM has been validated for sleep–wake detection (it shows higher sensitivity and specificity than actigraphy alone) compared to sleep diaries [73] and polysomnography (PSG) [74] and it can be used instead of DLMO (dim light melatonin onset) to predict the internal phase [80]. Besides, we have proven its usefulness in very different populations, such as night-shift workers [81, 82], babies [83, 84], hypertensive patients and patients with metabolic syndrome [85, 86], elderly population [84, 87], patients with mild cognitive impairment [88], or more recently in patients with sleep-disordered breathing [89], Parkinson’s disease [90, 91•], and cancer [61] (Table 1).

Therefore, this wearable technology opens the possibility to study, on a long-term basis, a wide range of patients under real-time conditions and thus providing, at the same time, information on a single individual that can then be used to guide health-related decisions.

## ACM for Circadian System Status Assessment in Cancer Patients: State of the Art

### Rest/Activity Rhythm

Rest/activity rhythm (RAR) is one of the most used biomarkers of CS functioning in cancer patients [92], as it can be non-invasively recorded for long periods by using a wrist-worn actigraphy sensor and is compatible with oncological treatments (Table 1).

In 2000, Mormont et al. [26] evaluated, in metastatic colorectal cancer patients, the predictive power of circadian rhythms’ robustness (an index of rhythm’s amplitude) over the patients’ prognosis. The subjects’ RAR was an independent prognostic factor for cancer patient’s survival (patients with poor circadian rhythmicity had a fivefold higher risk of dying within 2 years than patients with a better circadian rhythmicity) and tumor response as well as a quantitative indicator of QoL, better than other overt circadian rhythms such as plasma cortisol or leucocyte count. Since then, several oncological studies have shown that cancer patients exhibit a disruption of RAR before treatment [65, 71, 93] that worsens with chemotherapy [31, 36, 58, 94].

An unstable RAR associates to sleep impairment and insomnia [59, 63] and a subsequent decrease in QoL [22], which may negatively impact survival in cancer patients [23, 26, 65, 95]. Cancer patients’ RAR also experience a deterioration in robustness, which may be used, along with the rhythm stability, in future studies to illustrate clinicians as a potential prognostic factor [26, 62–64].

### Sleep/Wake Rhythm

Prevalence of sleeping disorders in modern society is continuously increasing (it affects up to 40–50% of the global population) [96], and this elevated incidence is enhanced in cancer patients (up to 60%). Cancer patients report sleeping disorders before, during, and after treatment, and usually persist in the long term [36, 63, 95, 97, 98]. Furthermore, low sleep quality is commonly associated with fatigue, psychological effects, such as anxiety or depression [62, 99, 100], revealing what has been called a cluster of cancer symptoms [24]. These symptoms interact with each other and can negatively impact the patient’s QoL, treatment efficacy, and survival [101].

Brzezinski determined that tumors alter existing rhythms and reset the SCN, which causes changes in the sleep/wake rhythm (SWR) [102]. Alternatively, one of the main causes of fatigue experienced by cancer patients during treatment, apart from the direct effects caused by cancer treatment itself, is related to the pro-inflammatory cytokine network activity. Cytokines rise inflammatory responses in the central nervous system (CNS), disrupt the hypothalamus–pituitary–adrenal axis and, consequently, the sleep/wake rhythm [27, 103, 104]. This affects SWR regularity and results in increased fatigue, insomnia, or depression caused by several SCN response mechanisms [64, 105]. Besides, severe physical symptoms (i.e., nocturia, dyspnea, or cough) have been reported in lung cancer patients under treatment who experience SWR disruption [106] and several studies indicate that a sleep/wake disruption may even increase the risk of death in cancer patients [22, 26, 27].

Liu et al. [33], based on Berger et al. [59] conclusions, remarked the need to perform longitudinal studies analyzing the relationship between sleep and health-related QoL (HQ-QoL) in cancer patients through objective methods for sleep assessment (Table 1). Komarzynski et al. compared sleep quality estimations from subjective methods (through questionnaires MDSAI (M.D. Anderson Symptom Inventory) and PROM (patient-reported outcome measures)) and from ACM (actigraphy) in advanced stage cancer patients undergoing chemotherapy, and observing that awakenings during the sleep period (estimated through ACM) correlated with subjective sleeping disorders complaints [72]. Therefore, the lower the number of night awakenings the best subjective sleep quality.

Frequent night awakenings or fragmented sleep not only impact negatively the subjective perception of sleep quality, but may also correlate with an altered innate immune function [107], lower natural killer cell count, and reduced survival [34]. Alternatively, a prolonged nocturnal sleep improves QoL, while longer sleep latencies and sleep interruptions worsen it [39]. Besides, a sleep efficiency (% real time of sleep related to total time in bed) lower than 85% is independently associated with lower survival in advanced breast cancer patients [108].

Chang and Lin analyzed changes on SWR, sleep quality, anxiety, depression, fatigue, and QoL caused before, during, and after treatment in lung cancer patients [38]. Results showed low SWR robustness before treatment. Compared to basal values, SWR significantly improved in week 48 after beginning the treatment, and anxiety significantly improved in weeks 6, 12, 24, and 40 after. In contrast, fatigue exacerbated in weeks 8 and 48. Furthermore, QoL significantly improved from week 6 until the end of treatment. QoL was negatively affected by poor sleep quality and depression symptoms while was positively impacted by a regular SWR.

Although sleep impairment and fatigue experienced by cancer patients are generally believed to be iatrogenic, partly caused by the psychological impact and the physiological consequences of anti-cancer treatments, further evidence is needed to determine its possible effect on tumorigenesis activation [109, 110]. In 2016, Erren and colleagues performed a meta-analysis of sleep and cancer incidence among more than 1,500,000 subjects from 13 countries and, although they failed to find a clear answer to the question of how cancer and sleep are connected in humans, authors concluded that a complex chronobiological relationship, presumably multidirectional, is plausible [111].

Garland et al. [112] suggested that a perturbation in sleep continuity associates to, or is consequence of, an already initiated disease and, as such, can represent a sign of yet-to-be-diagnosed tumors. It is known that night sleep enhances the immune system against any infection [107, 113]. A lack of sleep induces lower levels of pro-inflammatory cytokines [114, 115]. Nevertheless, sleep loss also activates the sympathetic nervous system (SNS) and, after a sleep disruption, the excess of adrenergic activity is believed to promote pro-inflammatory genes expression leading to increased diurnal levels of innate immunity and inflammation markers. There is a growing body of evidence pointing to inflammation as a key factor in cancer incidence and recurrence [107].

### Body Position and Temperature

In general, actigraphy has proved to be very sensitive for detecting sleep, but its ability to detect wake states during the night (specificity) is not so high [116, 117]. Actigraphy tends to overestimate sleep, as it considers motionless moments as sleep. That is the reason why it loses clinical importance when studying specific pathologies like insomnia, in which the subject lies awake for long periods with almost no movement [118, 119]. Furthermore, the movements of a bed partner, vehicle vibrations, and sensor removal can all affect the measurement reliability [120].

To counteract the inaccuracy associated to the use of one single variable, multivariable recordings under ambulatory conditions have recently been proposed to assess alterations of the circadian system and sleep [73, 121] (Table 1). These ACM procedures integrate a combination of variables, such as temperature, activity, and body position, which provide complementary information about the CS functionality.

Core body temperature (CBT) follows a circadian pattern which is generated by the SCN and thus, apart from reflecting the pacemaker function, provides relevant information about the inner phase and amplitude [61, 73, 78, 122]. Besides, CBT acts as an effector involved in the internal coordination of peripheral clocks, so as in clock-controlled signaling pathways in tumors [75, 120]. Based upon this knowledge, several authors point to body temperature monitoring as an appropriate indicator to personalize anti-cancer treatments [68]. However, its evaluation is not problem-free: the CBT rhythm can be determined using a rectal probe connected to an external recorder [122, 123] (a system that is neither safe nor convenient for ambulatory assessment of rhythms in cancer patients) or telemetric pills (which are very expensive and provide records that are too short) [61]. In 2008, Sarabia et al. showed that peripheral temperature rhythm, determined by a wireless data logger attached to the inner side of the wrist, was a robust rhythm with a pattern that is almost identically reverse to that of CBT [75]. Evidence suggests that sleepiness may be more closely linked to increased distal skin temperature (DST) than to a decrease in CBT [124, 125]. In fact, wrist temperature alone can discriminate between wake and sleep with high specificity and sensitivity and even between phases 1 + 2 and 3 [126], although it fails to identify sleep interruptions when they are too short. To date, DST has been used to evaluate circadian rhythms under several physiological and pathological conditions, such as newborn circadian maturation [127], mild cognitive impairment [74], metabolic syndrome and obesity [86] among others; and a correlation has been found with clock gene polymorphisms [128]. Furthermore, DST is highly correlated to the DLMO, considered to be the most robust circadian phase marker [80]. However, although peripheral temperature rhythm has been shown to exhibit a strong endogenous component, due to a relevant genetic influence [77, 129], it is subjected to environmental and physiological influences, including physical activity, body position, high environmental temperatures, and sleep itself [75, 130–132].

In 2010, the Chronobiology Lab from the University of Murcia (Spain) developed an integrated variable called TAP (Temperature, Activity, body Position), which is based on MCA-provided information of DST, motor activity, and body position rhythms. The overall aim of creating this integrated variable was to avoid problems related to masking (that is, the influence of external signals that affect or mask overt rhythms) in the recoding of single variables and thus to allow for a better assessment of the CS functioning [73]. Integrated variable TAP was validated as a useful tool for circadian patterns classification according to robustness. Body position, when the sensor is appropriately positioned, helps to depict daily habits and permits distinguishing when the subject is lying down or sitting (for example, at a computer), in spite of low activity levels in both cases [73]. Besides, integration of body position and motor activity to DST allows to identify sleep interruptions and to calculate several reliable sleep parameters, such as total time of sleep or sleep efficiency [73].

TAP algorithm has demonstrated its usefulness for RAR evaluation [80] and its values correlated very well with sleep logs from PSG, the “gold-standard” of sleep studies [74]. Results from Ortiz-Tudela et al. [74] show that integrated variable TAP is the most sensitive and specific to discriminate sleep from wakefulness and has higher coincidence rates with PSG than any other published actigraphy measurements [73, 116, 117]. Moreover, the simultaneous recording of multiple overt rhythms allows us to assess their internal synchrony, constituting a first approach to evaluate whether temporal order is maintained.

Finally, Ortiz-Tudela et al. [61] showed for the first time that the circadian system presents a huge variability in terms of phase distribution (12-h spread among subjects) in cancer patients and more importantly, a moderately perturbed internal temporal order. This study corroborated what previous research in cancer patients had shown: the existence of a perturbed RAR even before chemotherapy, which is negatively impacted by treatment [24, 36, 60, 70].

### Light Exposure

The use of light sensors as part of ACM allows to record environmental light exposure (intensity and spectrum) during 24 h and gives additional information to characterize the subjects’ life habits with no need of sleep diaries. Recently, Madrid-Navarro et al. [91•] integrated visible light exposure into the new variable TAPL (temperature, activity, position, and light), a modification of the TAP algorithm [73], for automatic detection of sleep and wake periods in Parkinson’s patients.

After a polysomnographic validation of sleep detection by ACM, TAPL was proven to be a useful tool for evaluating, while subjects develop their normal life, the main sleep parameters: time in bed, total sleep time, sleep efficiency, and wake after sleep onset (WASO), without the need to resort to different specific algorithms for each sleep pathology or age group [91•].

## Chronotherapy and Cancer: Past, Current, and Future Approaches to Cancer Treatment

Since the discovery of the biological clock, cancer treatments are administered at the appropriate time-of-day, according to biological rhythms. This way, chronotherapy has been shown to improve cancer treatment efficacy [133]. In the last two decades, different experimental and clinical studies have reported positive associations between the circadian clock and drug response in cancer patients, while others have reported a negative correlation between chronodisruption during and/or after cancer treatment with survival rate in cancer animal models and patients [134]. These findings suggest a role of the circadian clock in the outcome of cancer progression and treatment response, specifically in mechanisms of resistance to chemotherapy, controlled by the circadian clock [135–137], which regulates fundamental processes in the cell, often targeted machinery of cytotoxic anticancer drugs, as well as modulating the absorption, metabolism, and elimination of these drugs. Of note, several clinical studies have shown that adverse effects experienced by cancer patients treated with cisplatin-based chemotherapy decreases when applied in a chronomodulated context [138, 139], thus proving that chronomodulated drug administration provides the potential to optimize the dosage of drugs and duration of treatments to efficiently eliminate cancer cells while reducing adverse effects and prevent early drug resistance. Also, downregulation of core clock gene Per2 in tumors predicted poor survival in chemo-naive patients with metastatic colorectal cancer. This was the first report of an association between clock gene downregulation and outcome in any cancer [140]. In rectal cancer, a prospective single-arm phase II with capecitabine and radiotherapy treatment in 85 patients with oral administration of capecitabine (60% dose at 8 am and 40% dose at noon) obtained a total tumor regression in 20% of patients, with no grade IV toxicities reported [141]. Another study on advanced non-small cell lung carcinoma randomized 41 patients with cisplatin administration at 6 pm or at 6 am vs routine chemotherapy. Lower gastrointestinal toxicity (*p* < 0.05) and higher clearance for cisplatin administered at 6 pm (*p* < 0.05) were observed in the chronotherapy group [138]. Two clinical trials compared the toxicity of two dosing times of anthracyclines and cisplatin in patients with advanced ovarian cancer. Both randomized studies demonstrated that doxorubicin or theprubicin when administered around 6 am and cisplatin between 4 and 8 pm produced significantly fewer severe hematologic suppression and renal toxicity than when given 12 h earlier, respectively [142].

Although the clinical relevance of chronopharmacology principles in cancer is gradually maturing with the application of clinical big data [143], in vitro pharmacology, mobile platforms and apps [144], and mathematical modeling [145] to help expand our knowledge of the molecular clock and its relevance to human disease, some challenging issues facing chronotherapeutic approaches still remain to be highlighted (such as how a given cancer affects the clock and vice versa, how a given oncotherapy and other factors, such as our lifestyle and environment (personal chronotypes), affect the clock). This is probably due to variability of individual biological rhythms.

Therefore, optimizing the timing of drugs with a narrow therapeutic index and significant circadian fluctuation could lead to significant clinical benefit from improvements in efficacy and minimization of toxicity [146, 147]. The conception of novel anticancer drug delivery systems and the combination with chronobiology may provide a powerful tool to optimize cancer therapy and represents a new challenge to improve the quality of life and survival of cancer patients through personalized cancer chronotherapeutics.

## Conclusions

Although the results of many epidemiologic and animal studies are consistent with a role for the circadian clock in the genesis and progression of tumors, available data are still insufficient to conclude that clock disruption is generally carcinogenic. Nevertheless, both experimental and clinical data support the relevance of sleep and circadian rhythm disruption, or CD, in the expression and development of cancer. Besides, CD correlates with poor QoL and function. ACM, through wearable technology, opens the possibility to assess CD in cancer patients on a long-term basis, under real-time conditions, and provides, at the same time, objective information on a single individual that can then be used to guide health-related decisions.

ACM based on multi-variable recording continuously assesses circadian biomarkers, such as those provided by the rest/activity, body position, distal skin temperature, and light exposure monitoring computed in the integrated variable TAPL, and constitutes a unique tool to apply big-data analysis and design successful interventions in cancer patients, taking into account the status and phase of individual circadian systems. The great inter-patient variability at baseline, during and after treatment, and the differing profound effects of chemotherapy on circadian robustness, phase, and internal order synchronization confirm the interest of such multi-parametric evaluation of cancer outpatients.

The useful information provided by this concomitant TAPL monitoring is also relevant for interventional studies targeting the circadian timing system, in order to enhance or protect its function, with the aim of improving the wellbeing and outcomes of cancer patients. In this sense, ACM allows for an integrated analysis of the internal, external, and social times of the individual and thus helps to design individualized strategies to revert CD, first considering life habits (timing and regularity of bright light exposure, physical exercise, work duties, social and family life, sleep/wake routine, fasting/feeding schedule…), before addressing pharmacological approaches.

Thus, there is a certain biological plausibility that needs to be verified and studied, which could relate CD alterations and their impact on mechanisms and evolution of cancer. The objectivity that new technologies (wearables) can provide in their study needs to be further implemented in controlled and well-designed clinical trials in order to clarify and help to understand this relation. Likewise, it will be necessary to delve into the molecular mechanisms that may relate CD and tumorigenesis, especially in light of new treatments such as immunotherapy, where the tumor microenvironment is a determinant factor in the response. Future hypothesis-driven and discovery-oriented research should focus on specific interactions between clock components and carcinogenic mechanisms to realize the full clinical potential of the relationship between clocks and cancer.
